# The genome sequence of the Eurasian Curlew, *Numenius arquata *(Linnaeus, 1758)

**DOI:** 10.12688/wellcomeopenres.24272.1

**Published:** 2025-06-02

**Authors:** Grace Walsh, Barry O’ Donoghue, Jacob Höglund, Barry John McMahon, Wellcome Sanger Institute Tree of Life Management, Samples and Laboratory team, WellcomeSanger Institute Scientific Operations: Sequencing Operations, Wellcome Sanger Institute Tree of Life Core Informatics team, Tree of Life Core Informatics collective, Darwin Tree of Life Consortium

**Affiliations:** 1 UCD School of Agriculture and Food Science, Agriculture and Food Science, University College Dublin, Dublin, Leinster, Ireland; 2 National Parks and Wildlife Service, Dublin, Ireland; 3 Evolutionsbiologiskt centrum (EBC), Uppsala, Sweden

**Keywords:** Numenius arquata subspecies arquata, Eurasian Curlew, genome sequence, chromosomal, Charadriiformes

## Abstract

We present a genome assembly from a female specimen of *Numenius arquata* (Eurasian Curlew; Chordata; Aves; Charadriiformes; Scolopacidae). The assembly contains two haplotypes with total lengths of 1,348.86 megabases and 1,198.36 megabases. Most of haplotype 1 (89.99%) is scaffolded into 41 chromosomal pseudomolecules, including the W and Z sex chromosomes. Haplotype 2 was assembled to scaffold level. The mitochondrial genome has also been assembled, with a length of 17.13 kilobases. Gene annotation of this assembly on Ensembl identified 15,412 protein-coding genes.

## Species taxonomy

Eukaryota; Opisthokonta; Metazoa; Eumetazoa; Bilateria; Deuterostomia; Chordata; Craniata; Vertebrata; Gnathostomata; Teleostomi; Euteleostomi; Sarcopterygii; Dipnotetrapodomorpha; Tetrapoda; Amniota; Sauropsida; Sauria; Archelosauria; Archosauria; Dinosauria; Saurischia; Theropoda; Coelurosauria; Aves; Neognathae; Neoaves; Charadriiformes; Scolopacidae; *Numenius*; *Numenius arquata* (Linnaeus, 1758) (NCBI:txid31919)

## Background

**Figure 1.**
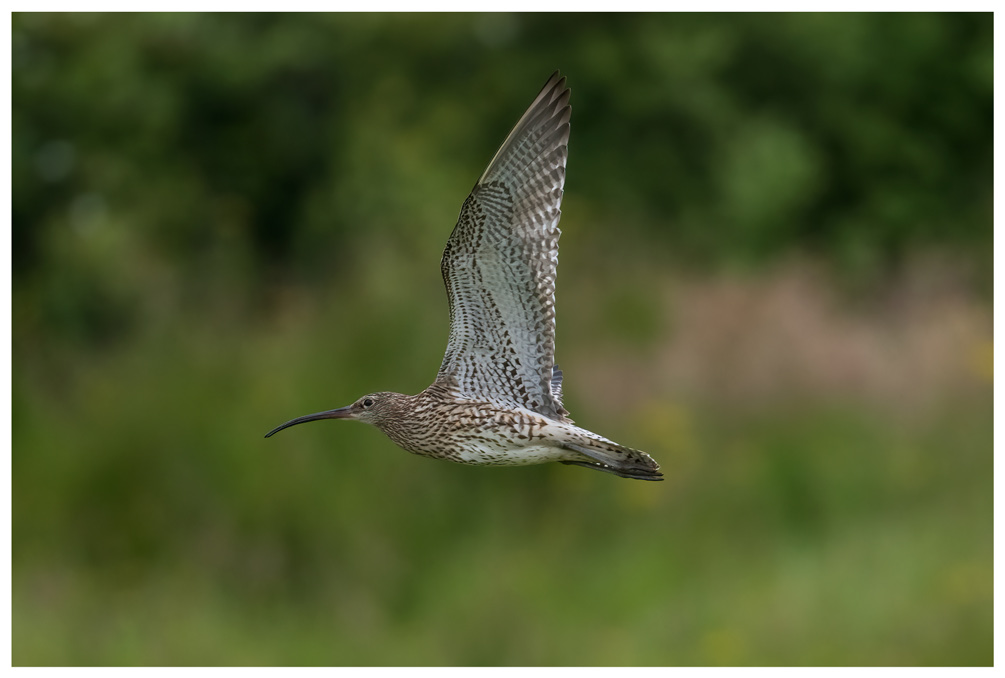
Eurasian Curlew (Numenius arquata) at its breeding grounds in Co. Monaghan, Ireland during June 2022. Credit: William O’Connor.

The Eurasian Curlew ( *Numenius arquata*) ( Figure 1) is Europe’s largest wading bird with a long downward curved bill and streaked brown plumage. It is sexually dimorphic, with females having a longer bill and larger body size ( van Duivendijk, 2024).

There are three subspecies – *N. a. arquata, N. a. orientalis and N. a. suschkini* – corresponding to glacial refugia ( Tan *et al.*, 2019). The nominate subspecies *N. a. arquata* is highly migratory, breeding from Ireland to the Ural Mountains, and Fennoscandia to Spain. It winters typically in coastal areas including Iceland, the British and Irish Isles, Mediterranean, Northeast Africa and into Western India ( Svensson *et al.*, 2022). The *N. a. orientalis* breeding population ranges east of the Ural Mountains across temperate Siberia. *N. a. suschkini* breeds south of the Urals in Russia and Kazakhstan ( BirdLife International, 2017b).

*N. a. arquata* (hereafter Curlew) breeds on the ground in open habitats such as raised and upland peatlands, improved agricultural grassland and arable fields. Curlew show high site fidelity, returning to the same breeding areas each year ( Schwemmer *et al.*, 2016; Taylor & Dodd, 2013). Migration to breeding grounds occurs from March/April. They form monogamous pairs, with females typically laying 3 to 4 eggs in shallow scrapes that are incubated by both parents. Chicks are precocial and will migrate to wintering grounds from June/July onwards and will not return to breed for 2 to 3 years. The length of the breeding period varies depending on latitude ( Pederson *et al.*, 2022).

The IUCN classifies Curlew as Near Threatened as of 2017 ( BirdLife International, 2017a) with an estimated global population of 610,000 to 830,000 ( Wetlands International, 2025). Finland holds 25% of this, with no major declines reported. However, serious declines are occurring in Russia and the UK, which hold 29% and 25% of the breeding population, respectively ( BirdLife International, 2021). High adult survival ( Pakanen & Kylmänen, 2023; Taylor & Dodd, 2013) indicates declines are driven by reduced breeding productivity. Threats include agricultural intensification ( Douglas *et al.*, 2014; Pearce-Higgins *et al.*, 2017), predation on eggs and chicks ( Brown, 2015; Roos *et al.*, 2018; Zielonka *et al.*, 2019) and land-use changes associated with agriculture and afforestation ( Brown, 2015; Douglas *et al.*, 2023).

Some Eurasian Curlew populations are on the brink of extinction ( Colhoun *et al.*, 2022; O’Donoghue *et al.*, 2019; Rodrigues *et al.*, 2019) and the species is facing many of the same threats as other ground-nesting birds ( McMahon *et al.*, 2020; Roos *et al.*, 2018). A chromosome-level reference genome will be valuable to conservation efforts. This specimen from the Irish population represents one of the most endangered cohorts of the species. This genome will facilitate further resequencing studies to assess genetic diversity, demographic history and migration. These insights can identify at-risk populations and guide management efforts to conserve this iconic species.

## Genome sequence report

### Sequencing data

The genome of a specimen of *Numenius arquata* ( Figure 1) was sequenced using Pacific Biosciences single-molecule HiFi long reads, generating 55.32 Gb from 6.66 million reads, which were used to assemble the genome. GenomeScope analysis estimated the haploid genome size at 1,179.93 Mb, with a heterozygosity of 0.48% and repeat content of 9.84%. These estimates guided expectations for the assembly. Based on the estimated genome size, the sequencing data provided approximately 45 coverage. Hi-C sequencing produced 85.60 Gb from 566.89 million reads, and was used to scaffold the assembly. RNA sequencing data were also generated and are available in public sequence repositories. Table 1 summarises the specimen and sequencing details.

**Table 1.** Specimen and sequencing data for *Numenius arquata*.

Project information			
**Study title**	Numenius arquata (Eurasian curlew)		
**Umbrella BioProject**	PRJEB71566		
**Species**	*Numenius arquata*		
**BioSpecimen**	SAMEA112693994		
**NCBI taxonomy ID**	31919		
Specimen information			
**Technology**	**ToLID**	**BioSample accession**	**Organism part**
**PacBio long read sequencing**	bNumArq3	SAMEA112694006	heart
**Hi-C sequencing**	bNumArq3	SAMEA112694006	heart
**RNA sequencing**	bNumArq3	SAMEA112694006	heart
Sequencing information			
**Platform**	**Run accession**	**Read count**	**Base count (Gb)**
**Hi-C Illumina NovaSeq 6000**	ERR12512739	5.67e+08	85.6
**PacBio Sequel IIe**	ERR12408794	1.62e+06	12.95
**PacBio Sequel IIe**	ERR12408795	2.24e+06	20.8
**PacBio Sequel IIe**	ERR12736857	2.80e+06	21.56
**RNA Illumina NovaSeq 6000**	ERR12512740	5.69e+07	8.59

### Assembly statistics

The genome was assembled into two haplotypes using Hi-C phasing. Haplotype 1 was curated to chromosome level, while haplotype 2 was assembled to scaffold level. The assembly was improved by manual curation, making 18 breaks and 64 and removing 76 haplotypic duplications. These interventions increased the total assembly length by 0.68% and decreased the scaffold count by 3.09%. The final assembly has a total length of 1,348.86 Mb in 1,818 scaffolds, with 785 gaps, and a scaffold N50 of 63.36 Mb ( Table 2).

**Table 2.** Genome assembly data for *Numenius arquata*.

Genome assembly	Haplotype 1	Haplotype 2
Assembly name	bNumArq3.hap1.1	bNumArq3.hap2.1
Assembly accession	GCA_964106895.1	GCA_964059295.1
Assembly level	chromosome	scaffold
Span (Mb)	1,348.86	1,198.36
Number of contigs	2,603	1,504
Number of scaffolds	1,818	811
Longest scaffold (Mb)	141.98	-
Assembly metrics (benchmark)	Haplotype 1	Haplotype 2
Contig N50 length (≥ 1 Mb)	2.61 Mb	2.82 Mb
Scaffold N50 length *(= chromosome N50)*	63.36 Mb	63.78 Mb
Consensus quality (QV) (≥ 40)	60.6	62.0
*k*-mer completeness	92.34%	86.01%
Combined *k*-mer completeness (≥ 95%)	99.67%	
BUSCO * (S > 90%; D < 5%)	C:97.3%[S:96.6%,D:0.7%], F:0.4%,M:2.3%,n:8,338	*-*
Percentage of assembly mapped to chromosomes (≥ 90%)	89.99%	-
Sex chromosomes (localised homologous pairs)	W and Z	-
Organelles (one complete allele)	Mitochondrial genome: 17.13 kb	*-*
Genome annotation of assembly GCA_964106895.1 at Ensembl		
Number of protein-coding genes	15,412	
Number of non-coding genes	858	
Number of gene transcripts	25,129	

* BUSCO scores based on the aves_odb10 BUSCO set using version 5.5.0. C = complete [S = single copy, D = duplicated], F = fragmented, M = missing, n = number of orthologues in comparison.

**Figure 2.**
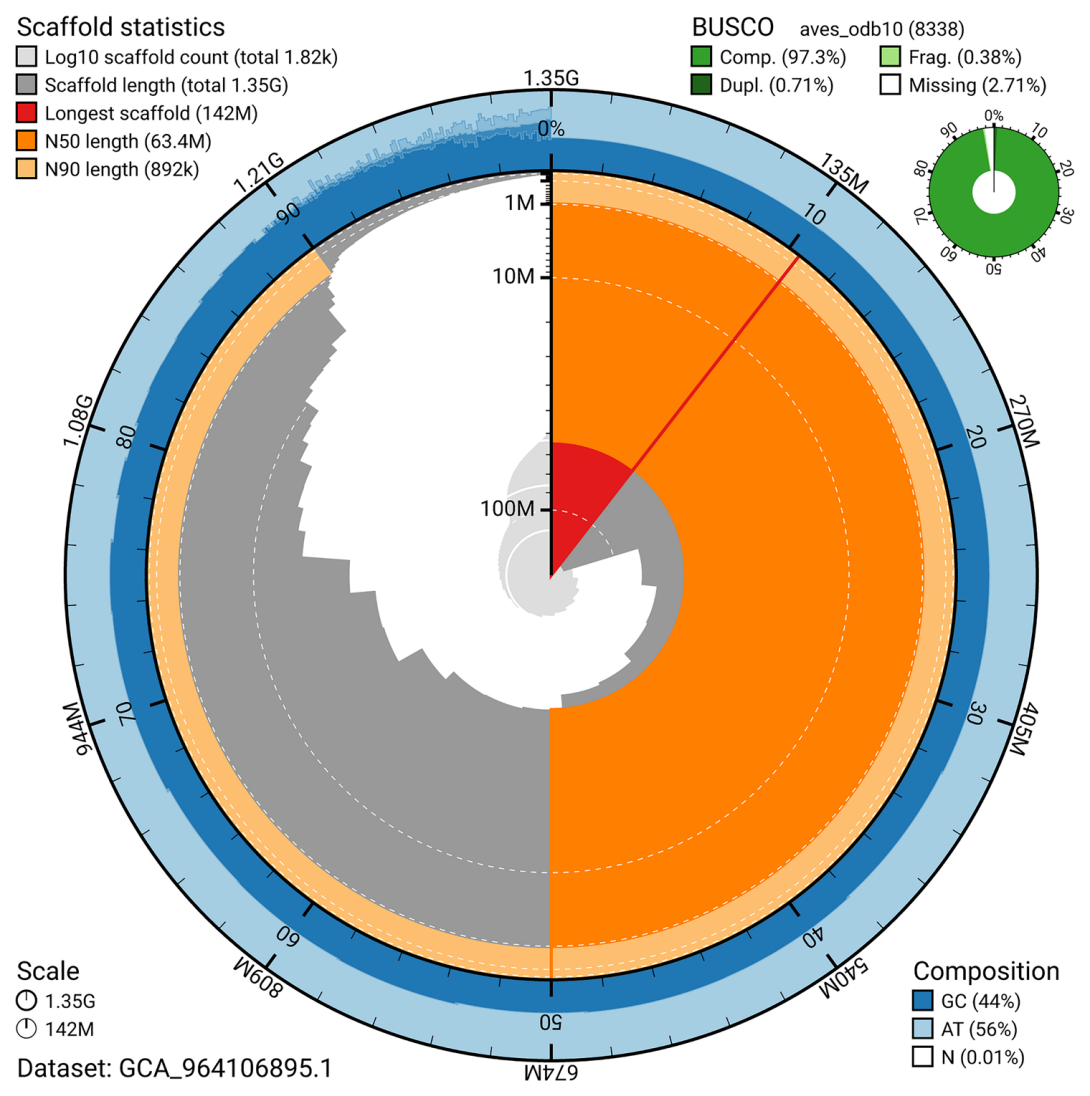
Genome assembly of *Numenius arquata*, bNumArq3.hap1.1: metrics. The BlobToolKit snail plot provides an overview of assembly metrics and BUSCO gene completeness. The circumference represents the length of the whole genome sequence, and the main plot is divided into 1,000 bins around the circumference. The outermost blue tracks display the distribution of GC, AT, and N percentages across the bins. Scaffolds are arranged clockwise from longest to shortest and are depicted in dark grey. The longest scaffold is indicated by the red arc, and the deeper orange and pale orange arcs represent the N50 and N90 lengths. A light grey spiral at the centre shows the cumulative scaffold count on a logarithmic scale. A summary of complete, fragmented, duplicated, and missing BUSCO genes in the aves_odb10 set is presented at the top right. An interactive version of this figure is available at https://blobtoolkit.genomehubs.org/view/GCA_964106895.1/dataset/GCA_964106895.1/snail.

**Figure 3.**
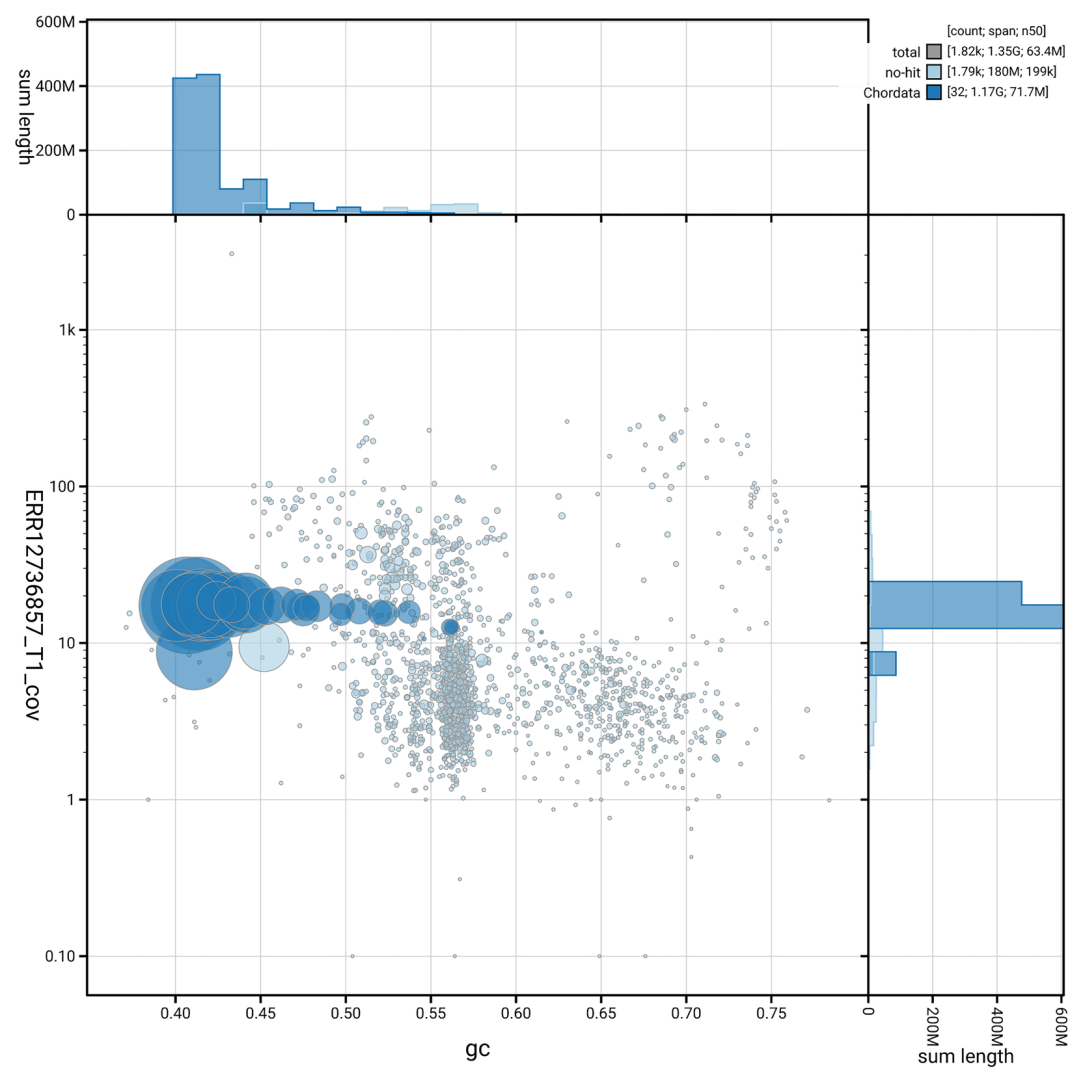
Genome assembly of *Numenius arquata*, bNumArq3.hap1.1: BlobToolKit GC-coverage plot. Blob plot showing sequence coverage (vertical axis) and GC content (horizontal axis). The circles represent scaffolds, with the size proportional to scaffold length and the colour representing phylum membership. The histograms along the axes display the total length of sequences distributed across different levels of coverage and GC content. An interactive version of this figure is available at https://blobtoolkit.genomehubs.org/view/GCA_964106895.1/dataset/GCA_964106895.1/blob.

**Figure 4.**
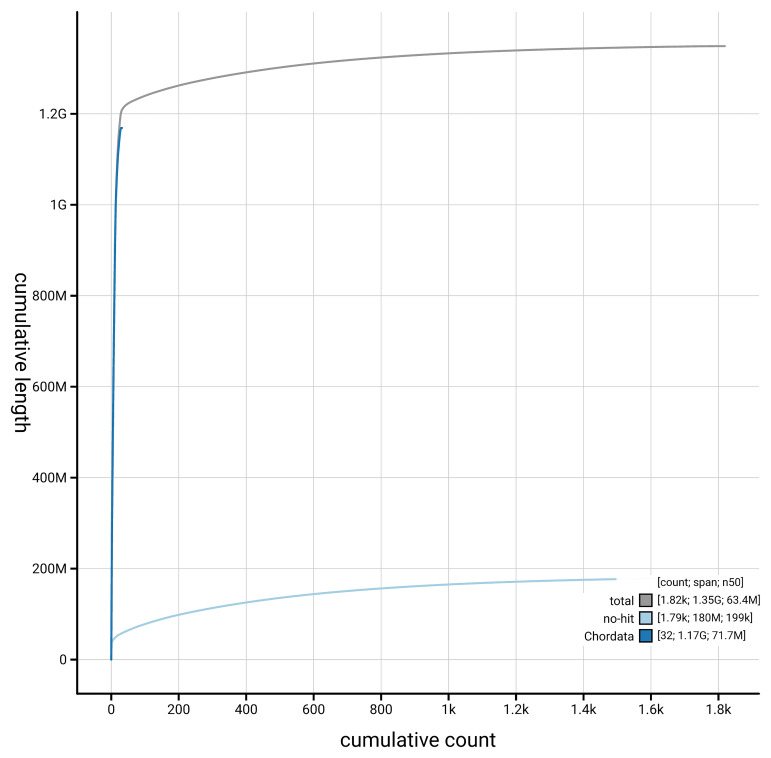
Genome assembly of *Numenius arquata,* bNumArq3.hap1.1: BlobToolKit cumulative sequence plot. The grey line shows cumulative length for all scaffolds. Coloured lines show cumulative lengths of scaffolds assigned to each phylum using the buscogenes taxrule. An interactive version of this figure is available at https://blobtoolkit.genomehubs.org/view/GCA_964106895.1/dataset/GCA_964106895.1/cumulative.

The snail plot in Figure 2 provides a summary of the assembly statistics, indicating the distribution of scaffold lengths and other assembly metrics. Figure 3 shows the distribution of scaffolds by GC proportion and coverage. Figure 4 presents a cumulative assembly plot, with separate curves representing different scaffold subsets assigned to various phyla, illustrating the completeness of the assembly.

**Figure 5.**
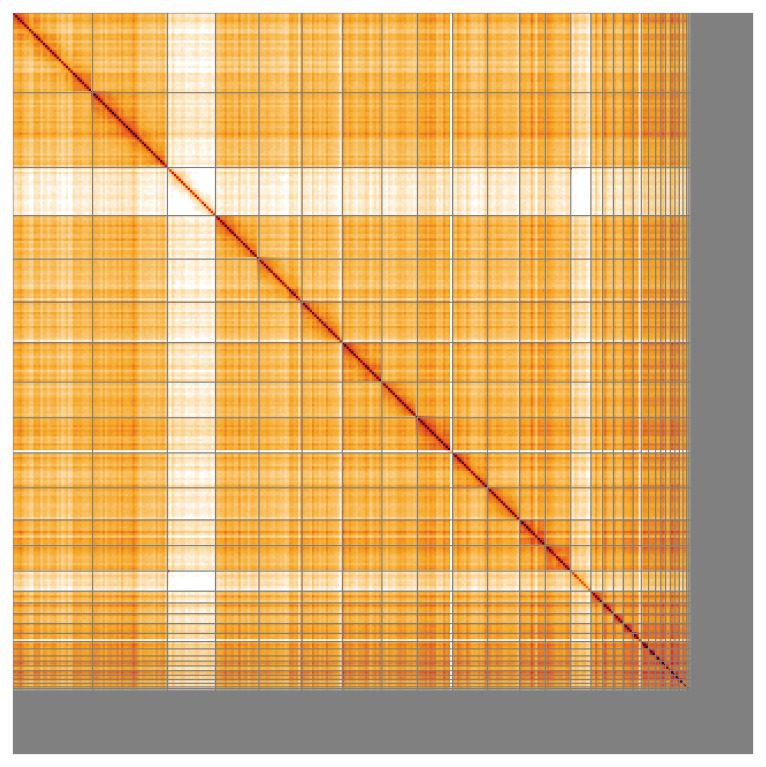
Genome assembly of *Numenius arquata:* Hi-C contact map of the bNumArq3.hap1.1 assembly, visualised using HiGlass. Chromosomes are shown in order of size from left to right and top to bottom. An interactive version of this figure may be viewed at https://genome-note-higlass.tol.sanger.ac.uk/l/?d=GCW9IHZKSY692hfEw8guvw.

Most of the assembly sequence (89.99%) was assigned to 41 chromosomal-level scaffolds, representing 39 autosomes and the W and Z sex chromosome. These chromosome-level scaffolds, confirmed by Hi-C data, are named according to size ( Figure 5; Table 3).

**Table 3.** Chromosomal pseudomolecules in the genome assembly of *Numenius arquata*, bNumArq3.

INSDC accession	Name	Length (Mb)	GC%
OZ067241.1	1	141.98	40.5
OZ067242.1	2	133.18	41.5
OZ067244.1	3	77.45	41.5
OZ067245.1	4	76.76	40
OZ067246.1	5	71.65	42
OZ067247.1	6	70.42	42
OZ067248.1	7	63.36	41
OZ067249.1	8	62.54	43
OZ067250.1	9	62.19	42
OZ067251.1	10	57.09	41
OZ067252.1	11	45.74	44
OZ067253.1	12	45.27	44
OZ067255.1	13	20.97	42.5
OZ067256.1	14	19.07	45.5
OZ067257.1	15	17.66	43.5
OZ067258.1	16	17.56	46
OZ067259.1	17	14.45	47.5
OZ067260.1	18	12.95	47
OZ067261.1	19	12.53	48
OZ067262.1	20	9.32	47.5
OZ067263.1	21	8.51	51
OZ067264.1	22	8.42	50
OZ067265.1	23	8.2	52.5
OZ067266.1	24	7.76	52
OZ067267.1	25	6.47	53.5
OZ067268.1	26	6.44	49.5
OZ067269.1	27	2.97	56
OZ067270.1	28	2.96	51.5
OZ067271.1	29	2.37	56
OZ067272.1	30	1.4	51
OZ067273.1	31	1.31	58
OZ067274.1	32	1.14	52.5
OZ067275.1	33	0.6	56
OZ067276.1	34	0.57	52.5
OZ067277.1	35	0.35	55
OZ067278.1	36	0.26	54.5
OZ067279.1	37	0.19	56
OZ067280.1	38	0.16	56
OZ067281.1	39	0.12	54.5
OZ067254.1	W	35.83	45
OZ067243.1	Z	85.6	41
OZ067282.1	MT	0.02	43.5

The mitochondrial genome was also assembled. This sequence is included as a contig in the multifasta file of the genome submission and as a standalone record.

### Assembly quality metrics

The estimated Quality Value (QV) and *k*-mer completeness metrics, along with BUSCO completeness scores, were calculated for each haplotype and the combined assembly. The QV reflects the base-level accuracy of the assembly, while *k*-mer completeness indicates the proportion of expected *k*-mers identified in the assembly. BUSCO scores provide a measure of completeness based on benchmarking universal single-copy orthologues.

For haplotype 1, the estimated QV is 60.6, and for haplotype 2, 62.0. When the two haplotypes are combined, the assembly achieves an estimated QV of 61.2. The *k*-mer recovery for haplotype 1 is 92.34%, and for haplotype 2 86.01%, while the combined haplotypes have a *k*-mer recovery of 99.67%. BUSCO v.5.5.0 analysis using the aves_odb10 reference set ( *n* = 8,338) identified 97.3% of the expected gene set (single = 96.6%, duplicated = 0.7%) for haplotype 1.

Table 2 provides assembly metric benchmarks adapted from Rhie *et al.* (2021) and the Earth BioGenome Project Report on Assembly Standards September 2024. The assembly achieves the EBP reference standard of **6.C.Q60**.

## Genome annotation report

The Numenius arquata genome assembly (GCA_964106895.1) was annotated externally by Ensembl at the European Bioinformatics Institute (EBI). This annotation includes 25,129 transcribed mRNAs from 15,412 protein-coding and 858 non-coding genes. The average transcript length is 32,918.42 bp. There are 1.54 coding transcripts per gene and 11.84 exons per transcript. For further information about the annotation, please refer to https://beta.ensembl.org/species/140581f7-4cba-4637-ac1d-486f8fc25b61.

## Methods

### Sample acquisition

The *Numenius arquata arquata* specimen used for genome sequencing (specimen ID SAN00002742, ToLID bNumArq3) was a juvenile that suffered an accidental death. Barry O’ Donoghue (Irish National Parks and Wildlife Service) collected the specimen from Co. Kerry, Ireland on 2022-07-16. The bird had been born that year in a captive rearing programme. The specimen was promptly frozen at –20°C. Once designated for sequencing it was transferred to a –80°C freezer on dry ice approximately 6 months after initial collection. Sampling was untaken by Grace Walsh (University College Dublin) and samples were preserved at –80°C before shipping on dry ice to the Wellcome Sanger Institute.

Metadata collection for samples adhered to the Darwin Tree of Life project standards described by Lawniczak *et al*. (2022).

### Nucleic acid extraction

The workflow for high molecular weight (HMW) DNA extraction at the Wellcome Sanger Institute (WSI) Tree of Life Core Laboratory includes a sequence of procedures: sample preparation and homogenisation, DNA extraction, fragmentation and purification. Detailed protocols are available on protocols.io ( Denton *et al.*, 2023). The bNumArq3 sample was prepared for DNA extraction by weighing and dissecting it on dry ice ( Jay *et al.*, 2023). Tissue from the heart was cryogenically disrupted using the Covaris cryoPREP ^®^ Automated Dry Pulverizer ( Narváez-Gómez *et al.*, 2023). HMW DNA was extracted using the MagAttract v2 protocol ( Oatley *et al.*, 2023a). DNA was sheared into an average fragment size of 12–20 kb in a Megaruptor 3 system ( Bates *et al.*, 2023). Sheared DNA was purified by solid-phase reversible immobilisation, using AMPure PB beads to eliminate shorter fragments and concentrate the DNA ( Oatley *et al.*, 2023b). The concentration of the sheared and purified DNA was assessed using a Nanodrop spectrophotometer and Qubit Fluorometer using the Qubit dsDNA High Sensitivity Assay kit. Fragment size distribution was evaluated by running the sample on the FemtoPulse system.

RNA was extracted from heart tissue of bNumArq3 in the Tree of Life Laboratory at the WSI using the RNA Extraction: Automated MagMax™ *mir*Vana protocol ( do Amaral *et al.*, 2023). The RNA concentration was assessed using a Nanodrop spectrophotometer and a Qubit Fluorometer using the Qubit RNA Broad-Range Assay kit. Analysis of the integrity of the RNA was done using the Agilent RNA 6000 Pico Kit and Eukaryotic Total RNA assay.

### Hi-C sample preparation and crosslinking

Hi-C data were generated from the heart of the bNumArq3 sample using the Arima-HiC v2 kit (Arima Genomics) with 20–50 mg of frozen tissue (stored at –80 °C). As per manufacturer’s instructions, tissue was fixed, and the DNA crosslinked using a TC buffer with 22% formaldehyde concentration, and a final formaldehyde concentration of 2%. The tissue was then homogenised using the Diagnocine Power Masher-II. The crosslinked DNA was digested using a restriction enzyme master mix, then biotinylated and ligated. A clean up was performed with SPRIselect beads prior to library preparation. DNA concentration was quantified using the Qubit Fluorometer v4.0 (Thermo Fisher Scientific) and Qubit HS Assay Kit, and sample biotinylation percentage was estimated using the Arima-HiC v2 QC beads.

### Library preparation and sequencing

Library preparation and sequencing were performed at the WSI Scientific Operations core.

***PacBio HiFi***

At a minimum, samples were required to have an average fragment size exceeding 8 kb and a total mass over 400 ng to proceed to the low-input SMRTbell Prep Kit 3.0 protocol (Pacific Biosciences), depending on genome size and sequencing depth required. Libraries were prepared using the SMRTbell Prep Kit 3.0 as per the manufacturer's instructions. The kit includes the reagents required for end repair/A-tailing, adapter ligation, post-ligation SMRTbell bead cleanup, and nuclease treatment. Size-selection and clean-up were carried out using diluted AMPure PB beads (Pacific Biosciences). DNA concentration was quantified using the Qubit Fluorometer v4.0 (ThermoFisher Scientific) with Qubit 1X dsDNA HS assay kit and the final library fragment size analysis was carried out using the Agilent Femto Pulse Automated Pulsed Field CE Instrument (Agilent Technologies) and the gDNA 55kb BAC analysis kit.

Samples were sequenced using the Sequel IIe system (Pacific Biosciences, California, USA). The concentration of the library loaded onto the Sequel IIe was in the range 40–135 pM. The SMRT link software, a PacBio web-based end-to-end workflow manager, was used to set-up and monitor the run, as well as perform primary and secondary analysis of the data upon completion.

***Hi-C***

For Hi-C library preparation, the biotinylated DNA constructs were fragmented using a Covaris E220 sonicator and size-selected to 400–600 bp using SPRISelect beads. DNA was then enriched using Arima-HiC v2 Enrichment beads. The NEBNext Ultra II DNA Library Prep Kit (New England Biolabs) was used for end repair, A-tailing, and adapter ligation, following a modified protocol in which library preparation is carried out while the DNA remains bound to the enrichment beads. PCR amplification was performed using KAPA HiFi HotStart mix and custom dual-indexed adapters (Integrated DNA Technologies) in a 96-well plate format. Depending on sample concentration and biotinylation percentage determined at the crosslinking stage, samples were amplified for 10–16 PCR cycles. Post-PCR clean-up was carried out using SPRISelect beads. The libraries were quantified using the Accuclear Ultra High Sensitivity dsDNA Standards Assay kit (Biotium) and normalised to 10 ng/μL before sequencing. Hi-C sequencing was performed on the Illumina NovaSeq 6000 instrument using 150 bp paired-end reads.

***RNA***

Poly(A) RNA-Seq libraries were constructed using the NEB Ultra II RNA Library Prep kit, following the manufacturer’s instructions. RNA sequencing was performed on the Illumina NovaSeq 6000 instrument.

### Genome assembly, curation and evaluation

***Assembly***

Prior to assembly of the PacBio HiFi reads, a database of *k*-mer counts ( *k* = 31) was generated from the filtered reads using FastK. GenomeScope2 ( Ranallo-Benavidez *et al.*, 2020) was used to analyse the *k*-mer frequency distributions, providing estimates of genome size, heterozygosity, and repeat content.

The HiFi reads were assembled using Hifiasm in Hi-C phasing mode ( Cheng *et al.*, 2021; Cheng *et al.*, 2022), resulting in a pair of haplotype-resolved assemblies. The Hi-C reads ( Rao *et al.*, 2014) were mapped to the primary contigs using bwa-mem2 ( Vasimuddin *et al.*, 2019). The contigs were further scaffolded using the provided Hi-C datain YaHS ( Zhou *et al.*, 2023) using the --break option for handling potential misassemblies. The scaffolded assemblies were evaluated using Gfastats ( Formenti *et al.*, 2022), BUSCO ( Manni *et al.*, 2021) and MERQURY.FK ( Rhie *et al.*, 2020).

The mitochondrial genome was assembled using MitoHiFi ( Uliano-Silva *et al.*, 2023), which runs MitoFinder ( Allio *et al.*, 2020) and uses these annotations to select the final mitochondrial contig and to ensure the general quality of the sequence.

***Assembly curation***

The assembly was decontaminated using the Assembly Screen for Cobionts and Contaminants (ASCC) pipeline. Flat files and maps used in curation were generated via the TreeVal pipeline ( Pointon *et al.*, 2023). Manual curation was conducted primarily in PretextView ( Harry, 2022) and HiGlass ( Kerpedjiev *et al.*, 2018), with additional insights provided by JBrowse2 ( Diesh *et al.*, 2023). Scaffolds were visually inspected and corrected as described by Howe *et al*. (2021). Any identified contamination, missed joins, and mis-joins were amended, and duplicate sequences were tagged and removed. The curation process is documented at https://gitlab.com/wtsi-grit/rapid-curation.

***Assembly quality assessment***

The Merqury.FK tool ( Rhie *et al.*, 2020), run in a Singularity container ( Kurtzer *et al.*, 2017), was used to evaluate *k*-mer completeness and assembly quality for both haplotypes using the *k*-mer databases ( *k* = 31) computed prior to genome assembly. The analysis outputs included assembly QV scores and completeness statistics.

A Hi-C contact map was produced for the final version of the assembly. The Hi-C reads were aligned using bwa-mem2 ( Vasimuddin *et al.*, 2019) and the alignment files were combined using SAMtools ( Danecek *et al.*, 2021). The Hi-C alignments were converted into a contact map using BEDTools ( Quinlan & Hall, 2010) and the Cooler tool suite ( Abdennur & Mirny, 2020). The contact map was visualised in HiGlass ( Kerpedjiev *et al.*, 2018).

The genome was analysed in the blobtoolkit pipeline, a Nextflow ( Di Tommaso *et al.*, 2017) port of the previous Snakemake Blobtoolkit pipeline ( Challis *et al.*, 2020). It aligns the PacBio reads in SAMtools ( Danecek *et al.*, 2021) and minimap2 ( Li, 2018) and generates coverage tracks for regions of fixed size. In parallel, it queries the GoaT database ( Challis *et al.*, 2023) to identify all matching BUSCO lineages to run BUSCO ( Manni *et al.*, 2021). For the three domain-level BUSCO lineages, the pipeline aligns the BUSCO genes to the UniProt Reference Proteomes database ( Bateman *et al.*, 2023) with DIAMOND blastp ( Buchfink *et al.*, 2021). The genome is also divided into chunks according to the density of the BUSCO genes from the closest taxonomic lineage, and each chunk is aligned to the UniProt Reference Proteomes database using DIAMOND blastx. Genome sequences without a hit are chunked using seqtk and aligned to the NT database with blastn ( Altschul *et al.*, 1990). The blobtools suite combines all these outputs into a blobdir for visualisation.

The blobtoolkit pipeline was developed using nf-core tooling ( Ewels *et al.*, 2020) and MultiQC ( Ewels *et al.*, 2016), relying on the Conda package manager, the Bioconda initiative ( Grüning *et al.*, 2018), the Biocontainers infrastructure ( da Veiga Leprevost *et al.*, 2017), as well as the Docker ( Merkel, 2014) and Singularity ( Kurtzer *et al.*, 2017) containerisation solutions.

Table 4 contains a list of relevant software tool versions and sources.

**Table 4.** Software tools: versions and sources.

Software tool	Version	Source
BEDTools	2.30.0	https://github.com/arq5x/bedtools2
BLAST	2.14.0	ftp://ftp.ncbi.nlm.nih.gov/blast/executables/blast+/
BlobToolKit	4.3.9	https://github.com/blobtoolkit/blobtoolkit
BUSCO	5.5.0	https://gitlab.com/ezlab/busco
bwa-mem2	2.2.1	https://github.com/bwa-mem2/bwa-mem2
Cooler	0.8.11	https://github.com/open2c/cooler
DIAMOND	2.1.8	https://github.com/bbuchfink/diamond
fasta_windows	0.2.4	https://github.com/tolkit/fasta_windows
FastK	666652151335353eef2fcd58880bcef5bc2928e1	https://github.com/thegenemyers/FASTK
GenomeScope2.0	2.0.1	https://github.com/tbenavi1/genomescope2.0
Gfastats	1.3.6	https://github.com/vgl-hub/gfastats
GoaT CLI	0.2.5	https://github.com/genomehubs/goat-cli
Hifiasm	0.19.8-r603	https://github.com/chhylp123/hifiasm
HiGlass	44086069ee7d4d3f6f3f0012569789ec138f42b84aa44357826c0b6753eb28de	https://github.com/higlass/higlass
MerquryFK	d00d98157618f4e8d1a9190026b19b471055b22e	https://github.com/thegenemyers/MERQURY.FK
Minimap2	2.24-r1122	https://github.com/lh3/minimap2
MitoHiFi	3	https://github.com/marcelauliano/MitoHiFi
MultiQC	1.14, 1.17, and 1.18	https://github.com/MultiQC/MultiQC
Nextflow	23.10.0	https://github.com/nextflow-io/nextflow
PretextView	0.2.5	https://github.com/sanger-tol/PretextView
samtools	1.19.2	https://github.com/samtools/samtools
sanger-tol/ascc	0.1.0	https://github.com/sanger-tol/ascc
sanger-tol/blobtoolkit	0.6.0	https://github.com/sanger-tol/blobtoolkit
Seqtk	1.3	https://github.com/lh3/seqtk
Singularity	3.9.0	https://github.com/sylabs/singularity
TreeVal	1.2.0	https://github.com/sanger-tol/treeval
YaHS	1.2a.2	https://github.com/c-zhou/yahs

### Wellcome Sanger Institute – Legal and Governance

The materials that have contributed to this genome note have been supplied by a Darwin Tree of Life Partner. The submission of materials by a Darwin Tree of Life Partner is subject to the **‘Darwin Tree of Life Project Sampling Code of Practice’**, which can be found in full on the Darwin Tree of Life website here. By agreeing with and signing up to the Sampling Code of Practice, the Darwin Tree of Life Partner agrees they will meet the legal and ethical requirements and standards set out within this document in respect of all samples acquired for, and supplied to, the Darwin Tree of Life Project.

Further, the Wellcome Sanger Institute employs a process whereby due diligence is carried out proportionate to the nature of the materials themselves, and the circumstances under which they have been/are to be collected and provided for use. The purpose of this is to address and mitigate any potential legal and/or ethical implications of receipt and use of the materials as part of the research project, and to ensure that in doing so we align with best practice wherever possible. The overarching areas of consideration are:

•     Ethical review of provenance and sourcing of the material

•     Legality of collection, transfer and use (national and international)

Each transfer of samples is further undertaken according to a Research Collaboration Agreement or Material Transfer Agreement entered into by the Darwin Tree of Life Partner, Genome Research Limited (operating as the Wellcome Sanger Institute), and in some circumstances other Darwin Tree of Life collaborators.

## Data Availability

European Nucleotide Archive: Numenius arquata (Eurasian curlew). Accession number PRJEB71566; https://identifiers.org/ena.embl/PRJEB71566. The genome sequence is released openly for reuse. The *Numenius arquata* genome sequencing initiative is part of the Darwin Tree of Life Project (PRJEB40665), the Sanger Institute Tree of Life Programme (PRJEB43745) and the Vertebrate Genomes Project (PRJNA489243). All raw sequence data and the assembly have been deposited in INSDC databases. Raw data and assembly accession identifiers are reported in Table 1 and Table 2.

## Author information

Members of the Wellcome Sanger Institute Tree of Life Management, Samples and Laboratory team are listed here: https://doi.org/10.5281/zenodo.12162482.

Members of Wellcome Sanger Institute Scientific Operations: Sequencing Operations are listed here: https://doi.org/10.5281/zenodo.12165051.

Members of the Wellcome Sanger Institute Tree of Life Core Informatics team are listed here: https://doi.org/10.5281/zenodo.12160324.

Members of the Tree of Life Core Informatics collective are listed here: https://doi.org/10.5281/zenodo.12205391.

Members of the Darwin Tree of Life Consortium are listed here: https://doi.org/10.5281/zenodo.4783558.
