# Supplementary material for: The genome sequence of the Eurasian Curlew, Numenius arquata (Linnaeus, 1758)
Source: Wellcome Open Res. 2025 Jun 2;10:298. [Version 1] doi: 10.12688/wellcomeopenres.24272.1 (PMC12381557; doi:10.12688/wellcomeopenres.24272.1)
Supplement: Peer review history

# Reviewer report: Reviewer response for version 1

**DOI:** 10.21956/wellcomeopenres.26769.r129257

**Published:** 2025-08-26

**Authors:** Natalie J Forsdick

**Affiliations:** 1 Manaaki Whenua Landcare Research, Lincoln, New Zealand

The manuscript 'The genome sequence of the Eurasian Curlew, *Numenius arquata* (Linnaeus, 1758)' describes the genome assembly and annotation for the Eurasian Curlew. As per the Darwin Tree of Life protocols, the assembly presented here was generated following the standard methods pipelines and reporting, and is of high quality as assessed against the standard benchmarks, achieving EBP 6.C.Q60. 

 I have a small number of minor suggestions/questions for the authors, as follows.

 The data in the ENA does not seem to be available as yet at https://identifiers.org/ena.embl/PRJEB71566.

 In the Sequencing data section, do the authors mean 45 **X** coverage?

 In the section Assembly statistics, it seems that a word is missing in 'making 18 breaks and 64 [???] and removing 76 haplotypic duplications'.

 There is a minor typo of 'datain' in the Genome assembly, curation and evaluation - Assembly section.

Are sufficient details of methods and materials provided to allow replication by others?

Yes

Is the rationale for creating the dataset(s) clearly described?

Yes

Are the datasets clearly presented in a useable and accessible format?

Yes

Are the protocols appropriate and is the work technically sound?

Yes

Reviewer Expertise:

Conservation genomics

I confirm that I have read this submission and believe that I have an appropriate level of expertise to confirm that it is of an acceptable scientific standard.

# Reviewer report: Reviewer response for version 1

**DOI:** 10.21956/wellcomeopenres.26769.r126305

**Published:** 2025-07-03

**Authors:** Annabel Whibley

**Affiliations:** 1 Bragato Research Institute, Clyde, New Zealand

Walsh and colleagues report the genome assembly and genome annotation of the Eurasian Curlew ( *Numenius arquata). * The introduction sets out the natural history of the species, subspecies delineation and the Near-Threatened conservation status of the species. The assembly is presented as two phased haplotypes, with Haplotype 1 curated to chromosome-level and Haplotype 2 to scaffold level.

 The Data Note follows Darwin Tree of Life project protocols, assembly and annotation pipelines and reporting templates. Appropriate methods are used, metadata is comprehensive and public accession links are active. The quality of the assembly is high (reaching EBP 6.C.Q60).

 The annotation includes evidence from RNAseq data obtained from heart tissue (also the source of the genomic sequence). I would query the reporting of the average transcript length as >32kb. This seems unfeasibly high. Could the meaning of this statistic be clarified in the template?

Are sufficient details of methods and materials provided to allow replication by others?

Yes

Is the rationale for creating the dataset(s) clearly described?

Yes

Are the datasets clearly presented in a useable and accessible format?

Yes

Are the protocols appropriate and is the work technically sound?

Yes

Reviewer Expertise:

Genomics, Bioinformatics, Evolution

I confirm that I have read this submission and believe that I have an appropriate level of expertise to confirm that it is of an acceptable scientific standard.
